# Food allergy is an important diseases associated to fibromyalgia

**DOI:** 10.1186/2045-7022-3-S3-P120

**Published:** 2013-07-25

**Authors:** FA Puccio, R Rojas, I Mosquera, A Hernandez, R Mosquera, L Jaua, M Lizarrale, D Cifarrelli, R Reyes

**Affiliations:** 1 Lab Inmunopatologia, Instituto de Biomedicina UCV, Caracas, Bolivarian Republic of Venezuela; 2 Unidad de Inmunoalergia, Caracas, Bolivarian Republic of Venezuela; 3 Instituto de Neurologia y Neurosciencias Aplicadas, Caracas, Bolivarian Republic of Venezuela

## Background

It has been proposed that allergy may be considered as one of the common associated conditions, like irritable bowel syndrome or migraine in fibromyalgia. Fibromyalgia is a clinical entity classified by the presence of chronic widespread pain in at least 11 of the 18 specified tender points and associated with symptoms like fatigue, irritable bowel, sleep disorder, chronic headaches, jaw pain, cognitive or memory impairment, etc.The aim of this study was to evaluate the presences of food allergy symptoms in Fibromyalgia patients.

## Methods

We evaluated 92 FM patients who assisted to Neurological and Neurosciences Institute, Caracas, Venezuela, by a multidisciplinary team work, we performed physical exam including ACR criteria for fibromyalgia diagnosis, neurological and cognitive evaluation, Hamilton depression and anxiety scales. Full clinical evaluation was performed and patient were classified according to the criteria of the International Consensus Report as suffering rhinitis only, asthma without significant rhinitis, and combined asthma and rhinitis as defined by the guidelines of the WHO/NIH Global Initiative for Asthma (GINA). Patients with atopic dermatitis were classified following Hanifin and Rajka. Food allergy induces symptoms were evaluated in a medical history and physical examination according to NIAD (Guidelines for the Diagnosis and Managementof Food Allergy). Skin prick tests (SPT) were performed with a battery of commercial allergens (ALK-Abello) extracts according to international guidelines. Specific IgE to common food allergens were detected.

## Results

We found that 61.50% of patients had rhinitis, 8.77% asthma and food allergy was present in 49% of cases. We also found that 50.87% of patient had gastric reflux symptom and 52.63 had gastritis. 66% of patients reports different symptoms with milk, orange and wheat. Specific milk proteins IgE were positive in a 50% of patients with gastrointestinal symptoms.

## Conclusion

Allergy condition and food allergy to common food, were important features to evaluate in pain and gastric associated diseases as Fibromyalgia, in order to improve better treatment to the patients.

## Disclosure of interest

None declared.
